# 2-(4-Chloro­phen­yl)-2-oxoethyl 4-hy­droxy­benzoate

**DOI:** 10.1107/S1600536811037500

**Published:** 2011-09-17

**Authors:** Hoong-Kun Fun, Tara Shahani, B. Garudachari, Arun M. Isloor, Kammasandra N. Shivananda

**Affiliations:** a X-ray Crystallography Unit, School of Physics, Universiti Sains Malaysia, 11800 USM, Penang, Malaysia; b Medicinal Chemistry Division, Department of Chemistry, National Institute of Technology-Karnataka, Surathkal, Mangalore 575 025, India; c Schulich Faculty of Chemistry, Technion Israel Institute of Technology, Haifa, Israel 32000

## Abstract

The title compound, C_15_H_11_ClO_4_, consists of a chloro­benzene ring and a phenol ring which are linked together by a 1,4-dioxo-2-oxabutane-1,4-diyl group. The dihedral angle between the chloro­benzene and phenol rings is 65.70 (11)°. In the crystal, inter­molecular O—H⋯O hydrogen bonds link the mol­ecules into chains along [010].

## Related literature

For background to phenacyl benzoate, see: Sheehan & Umezaw (1973 ▶); Gandhi *et al.* (1995 ▶); Huang *et al.* (1996 ▶); Ruzicka *et al.* (2002 ▶); Litera *et al.* (2006 ▶). For bond-length data, see: Allen *et al.* (1987 ▶).
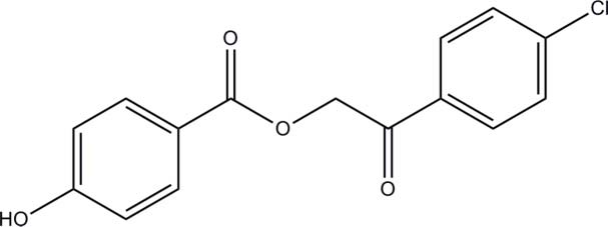


## Experimental

### 

#### Crystal data

C_15_H_11_ClO_4_
*M* *_r_* = 290.69
Monoclinic, 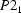

*a* = 5.5307 (10) Å
*b* = 8.1324 (15) Å
*c* = 14.857 (2) Å
β = 95.120 (4)°
*V* = 665.57 (19) Å^3^
*Z* = 2
Mo *K*α radiation
μ = 0.30 mm^−1^
*T* = 296 K
0.56 × 0.23 × 0.07 mm

#### Data collection

Bruker APEXII DUO CCD area-detector diffractometer
Absorption correction: multi-scan (*SADABS*; Bruker, 2009 ▶) *T*_min_ = 0.852, *T*_max_ = 0.981
6399 measured reflections
3571 independent reflections
2457 reflections with *I* > 2σ(*I*)
*R*_int_ = 0.027

#### Refinement

*R*[*F* ^2^ > 2σ(*F* ^2^)] = 0.044
*wR*(*F* ^2^) = 0.106
*S* = 1.02
3571 reflections
186 parameters
1 restraint
H atoms treated by a mixture of independent and constrained refinement
Δρ_max_ = 0.20 e Å^−3^
Δρ_min_ = −0.21 e Å^−3^
Absolute structure: Flack (1983 ▶), 1511 Friedel pairs
Flack parameter: −0.20 (8)

### 

Data collection: *APEX2* (Bruker, 2009 ▶); cell refinement: *SAINT* (Bruker, 2009 ▶); data reduction: *SAINT*; program(s) used to solve structure: *SHELXTL* (Sheldrick, 2008 ▶); program(s) used to refine structure: *SHELXTL*; molecular graphics: *SHELXTL*; software used to prepare material for publication: *SHELXTL* and *PLATON* (Spek, 2009 ▶).

## Supplementary Material

Crystal structure: contains datablock(s) global, I. DOI: 10.1107/S1600536811037500/bq2305sup1.cif

Structure factors: contains datablock(s) I. DOI: 10.1107/S1600536811037500/bq2305Isup2.hkl

Supplementary material file. DOI: 10.1107/S1600536811037500/bq2305Isup3.cml

Additional supplementary materials:  crystallographic information; 3D view; checkCIF report

## Figures and Tables

**Table 1:** Hydrogen-bond geometry (Å, °)

*D*—H⋯*A*	*D*—H	H⋯*A*	*D*⋯*A*	*D*—H⋯*A*
O4—H1*O*4⋯O3^i^	0.78 (4)	2.01 (4)	2.783 (3)	168 (3)

Symmetry code: (i) 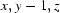.

**Fig. 1.**
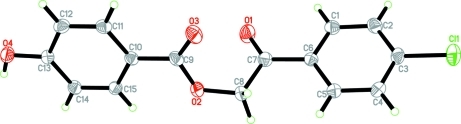
The molecular structure of the title compound, showing 30% probability displacement ellipsoids.

**Fig. 2.**
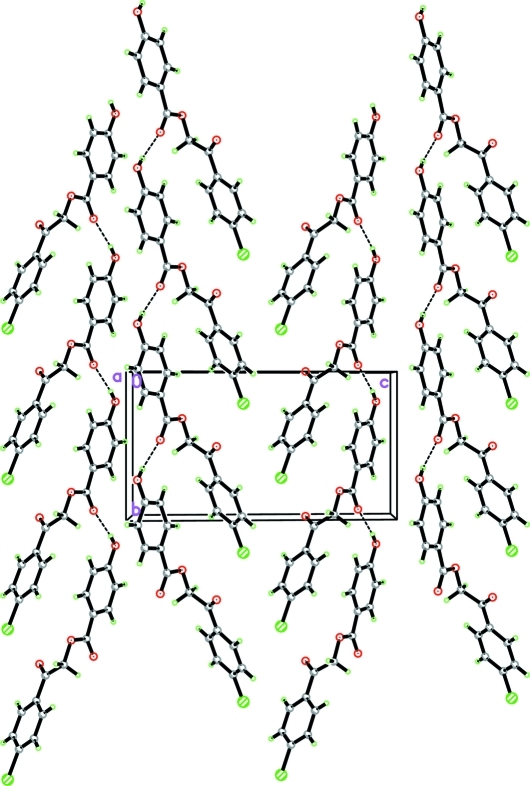
The crystal packing of the title compound, viewed along the a axis, showing a one-dimensional chain along [010].

## supplementary crystallographic information

### Comment

Phenacyl benzoate is a derivative of an acid formed by reaction between an acid and phenacyl bromide. These compounds find applications in the field of synthetic chemistry (Huang *et al.*, 1996; Gandhi *et al.*, 1995) such as in the synthesis of oxazoles, imidazoles, and benzoxazepines. They are also useful for photo-removable protecting groups for carboxylic acids in organic synthesis and biochemistry (Ruzicka *et al.*, 2002; Litera *et al.*, 2006; Sheehan & Umezaw, 1973). Keeping this in view, the title compound was synthesized to study its crystal structure.

The title compound, (Fig. 1), consists of a chlorobenzene (Cl1/C1–C6) ring and a phenol(O4/C10–C15) ring which are linked together by a 2-oxopropyl acetate (C7–C9/O1–O3) group. The dihedral angle formed between the chlorobenzene and a phenol ring is 65.70 (11) °. The bond lengths (Allen *et al.*, 1987) and angles are within normal ranges.

In the crystal packing (Fig. 2), intermolecular O4—H1O4···O3 hydrogen bonds (Table 1) link the molecules in one-dimensional chains along [010].

### Experimental

The mixture of 4-hydroxybenzoic acid (1.0 g, 0.0072 mol), potassium carbonate (1.10 g, 0.0079 mol) and 2-bromo-1-(4-chlorophenyl)ethanone (1.68 g, 0.0072 mol) in dimethylformamide (10 ml) was stirred at room temperature for 2 h. On cooling, colorless needle-shaped 2-(4-chlorophenyl)-2-oxoethyl 4-hydroxybenzoate begin to separate out. It was collected by filtration and recrystallized from ethanol. Yield: 1.95 g, 92.8%, M.p: 453–454 K.

### Refinement

The hydrogen atoms bound to C atoms were positioned geometrically [C–H = 0.9300–0.9700 Å] with *U*_iso_(H) = 1.2. The hydrogen atoms attached to the O atom was located from the difference map and refined freely, [O–H = 0.79 (3) Å].

### Crystal data

**Table:**

C_15_H_11_ClO_4_	*F*(000) = 300
*M**_r_* = 290.69	*D*_x_ = 1.450 Mg m^−^^3^
Monoclinic, *P*2_1_	Mo *K*α radiation, λ = 0.71073 Å
Hall symbol: P 2yb	Cell parameters from 1832 reflections
*a* = 5.5307 (10) Å	θ = 3.7–24.1°
*b* = 8.1324 (15) Å	µ = 0.30 mm^−^^1^
*c* = 14.857 (2) Å	*T* = 296 K
β = 95.120 (4)°	Plate, colourless
*V* = 665.57 (19) Å^3^	0.56 × 0.23 × 0.07 mm
*Z* = 2	

### Data collection

**Table:**

Bruker APEXII DUO CCD area-detector diffractometer	3571 independent reflections
Radiation source: fine-focus sealed tube	2457 reflections with *I* > 2σ(*I*)
graphite	*R*_int_ = 0.027
φ and ω scans	θ_max_ = 30.0°, θ_min_ = 2.8°
Absorption correction: multi-scan (*SADABS*; Bruker, 2009)	*h* = −5→7
*T*_min_ = 0.852, *T*_max_ = 0.981	*k* = −11→11
6399 measured reflections	*l* = −20→20

### Refinement

**Table:**

Refinement on *F*^2^	Secondary atom site location: difference Fourier map
Least-squares matrix: full	Hydrogen site location: inferred from neighbouring sites
*R*[*F*^2^ > 2σ(*F*^2^)] = 0.044	H atoms treated by a mixture of independent and constrained refinement
*wR*(*F*^2^) = 0.106	*w* = 1/[σ^2^(*F*_o_^2^) + (0.0346*P*)^2^ + 0.0709*P*] where *P* = (*F*_o_^2^ + 2*F*_c_^2^)/3
*S* = 1.02	(Δ/σ)_max_ = 0.001
3571 reflections	Δρ_max_ = 0.20 e Å^−^^3^
186 parameters	Δρ_min_ = −0.21 e Å^−^^3^
1 restraint	Absolute structure: Flack (1983), 1511 Friedel pairs
Primary atom site location: structure-invariant direct methods	Flack parameter: −0.20 (8)

### Special details

**Table:**

Geometry. All e.s.d.'s (except the e.s.d. in the dihedral angle between two l.s. planes) are estimated using the full covariance matrix. The cell e.s.d.'s are taken into account individually in the estimation of e.s.d.'s in distances, angles and torsion angles; correlations between e.s.d.'s in cell parameters are only used when they are defined by crystal symmetry. An approximate (isotropic) treatment of cell e.s.d.'s is used for estimating e.s.d.'s involving l.s. planes.
Refinement. Refinement of *F*^2^ against ALL reflections. The weighted *R*-factor *wR* and goodness of fit *S* are based on *F*^2^, conventional *R*-factors *R* are based on *F*, with *F* set to zero for negative *F*^2^. The threshold expression of *F*^2^ > σ(*F*^2^) is used only for calculating *R*-factors(gt) *etc*. and is not relevant to the choice of reflections for refinement. *R*-factors based on *F*^2^ are statistically about twice as large as those based on *F*, and *R*- factors based on ALL data will be even larger.

### Fractional atomic coordinates and isotropic or equivalent isotropic displacement parameters (Å^2^)

**Table:**

	*x*	*y*	*z*	*U*_iso_*/*U*_eq_	
Cl1	0.97678 (16)	1.71550 (10)	0.56971 (5)	0.0916 (3)	
O1	0.4790 (3)	0.9949 (3)	0.66410 (13)	0.0797 (5)	
O2	0.7288 (3)	0.81404 (19)	0.79114 (12)	0.0616 (4)	
O3	0.4559 (4)	0.95006 (19)	0.86448 (13)	0.0700 (5)	
O4	0.2024 (3)	0.2039 (2)	0.93940 (13)	0.0633 (4)	
C1	0.6002 (4)	1.3072 (3)	0.60310 (14)	0.0559 (5)	
H1A	0.4508	1.2636	0.5815	0.067*	
C2	0.6676 (5)	1.4603 (4)	0.57413 (16)	0.0634 (6)	
H2A	0.5650	1.5196	0.5331	0.076*	
C3	0.8875 (5)	1.5239 (3)	0.60661 (15)	0.0579 (6)	
C4	1.0422 (4)	1.4387 (3)	0.66746 (16)	0.0587 (6)	
H4A	1.1905	1.4837	0.6893	0.070*	
C5	0.9738 (4)	1.2856 (3)	0.69547 (15)	0.0548 (5)	
H5A	1.0778	1.2268	0.7362	0.066*	
C6	0.7520 (4)	1.2176 (3)	0.66383 (12)	0.0466 (4)	
C7	0.6694 (4)	1.0548 (3)	0.69417 (15)	0.0544 (5)	
C8	0.8296 (4)	0.9684 (3)	0.76653 (18)	0.0609 (6)	
H8A	0.9879	0.9496	0.7451	0.073*	
H8B	0.8517	1.0382	0.8195	0.073*	
C9	0.5374 (4)	0.8211 (3)	0.84121 (15)	0.0521 (5)	
C10	0.4532 (4)	0.6572 (3)	0.86395 (13)	0.0458 (5)	
C11	0.2512 (4)	0.6426 (3)	0.91413 (14)	0.0535 (5)	
H11A	0.1713	0.7367	0.9312	0.064*	
C12	0.1709 (4)	0.4916 (3)	0.93820 (14)	0.0565 (6)	
H12A	0.0353	0.4834	0.9707	0.068*	
C13	0.2901 (4)	0.3505 (3)	0.91444 (14)	0.0467 (5)	
C14	0.4930 (4)	0.3639 (3)	0.86646 (15)	0.0520 (5)	
H14A	0.5763	0.2698	0.8516	0.062*	
C15	0.5711 (4)	0.5153 (3)	0.84087 (15)	0.0495 (5)	
H15A	0.7052	0.5229	0.8075	0.059*	
H1O4	0.289 (6)	0.135 (5)	0.924 (2)	0.083 (11)*	

### Atomic displacement parameters (Å^2^)

**Table:**

	*U*^11^	*U*^22^	*U*^33^	*U*^12^	*U*^13^	*U*^23^
Cl1	0.1111 (6)	0.0700 (4)	0.0958 (5)	−0.0168 (4)	0.0211 (4)	0.0258 (4)
O1	0.0787 (12)	0.0704 (12)	0.0853 (11)	−0.0250 (11)	−0.0197 (9)	0.0065 (11)
O2	0.0618 (9)	0.0398 (8)	0.0840 (10)	0.0020 (8)	0.0114 (8)	0.0072 (9)
O3	0.0857 (12)	0.0393 (9)	0.0855 (12)	0.0051 (9)	0.0107 (10)	−0.0103 (9)
O4	0.0671 (10)	0.0527 (10)	0.0728 (10)	−0.0026 (10)	0.0207 (8)	0.0070 (9)
C1	0.0540 (12)	0.0605 (15)	0.0527 (11)	0.0018 (12)	0.0012 (9)	0.0002 (12)
C2	0.0657 (14)	0.0673 (16)	0.0562 (12)	0.0059 (13)	0.0010 (11)	0.0138 (12)
C3	0.0674 (14)	0.0545 (13)	0.0546 (11)	−0.0056 (12)	0.0208 (10)	0.0081 (11)
C4	0.0508 (12)	0.0629 (15)	0.0633 (13)	−0.0090 (12)	0.0096 (10)	−0.0003 (12)
C5	0.0509 (11)	0.0545 (13)	0.0587 (12)	−0.0006 (11)	0.0032 (9)	0.0043 (11)
C6	0.0480 (10)	0.0479 (11)	0.0444 (9)	0.0032 (10)	0.0066 (8)	−0.0032 (10)
C7	0.0560 (12)	0.0485 (12)	0.0582 (12)	−0.0018 (11)	0.0018 (10)	−0.0061 (11)
C8	0.0563 (12)	0.0468 (13)	0.0788 (15)	−0.0019 (11)	0.0014 (11)	0.0101 (12)
C9	0.0566 (12)	0.0415 (12)	0.0567 (12)	0.0036 (11)	−0.0031 (10)	−0.0033 (11)
C10	0.0485 (10)	0.0385 (10)	0.0499 (11)	0.0024 (9)	0.0009 (8)	−0.0027 (9)
C11	0.0536 (12)	0.0481 (12)	0.0595 (12)	0.0093 (10)	0.0085 (10)	−0.0084 (11)
C12	0.0519 (12)	0.0622 (15)	0.0568 (12)	0.0049 (12)	0.0128 (9)	−0.0031 (12)
C13	0.0510 (11)	0.0441 (11)	0.0451 (10)	−0.0010 (10)	0.0052 (9)	0.0012 (9)
C14	0.0580 (12)	0.0401 (12)	0.0594 (12)	0.0063 (10)	0.0128 (10)	−0.0010 (10)
C15	0.0489 (11)	0.0435 (11)	0.0569 (11)	0.0025 (11)	0.0101 (9)	−0.0013 (11)

### Geometric parameters (Å, °)

**Table:**

Cl1—C3	1.738 (3)	C5—H5A	0.9300
O1—C7	1.209 (3)	C6—C7	1.483 (3)
O2—C9	1.349 (3)	C7—C8	1.505 (3)
O2—C8	1.434 (3)	C8—H8A	0.9700
O3—C9	1.204 (3)	C8—H8B	0.9700
O4—C13	1.351 (3)	C9—C10	1.461 (3)
O4—H1O4	0.79 (3)	C10—C15	1.384 (3)
C1—C2	1.380 (4)	C10—C11	1.402 (3)
C1—C6	1.383 (3)	C11—C12	1.364 (4)
C1—H1A	0.9300	C11—H11A	0.9300
C2—C3	1.369 (4)	C12—C13	1.385 (3)
C2—H2A	0.9300	C12—H12A	0.9300
C3—C4	1.375 (4)	C13—C14	1.386 (3)
C4—C5	1.376 (3)	C14—C15	1.370 (3)
C4—H4A	0.9300	C14—H14A	0.9300
C5—C6	1.389 (3)	C15—H15A	0.9300
			
C9—O2—C8	116.48 (19)	C7—C8—H8A	109.2
C13—O4—H1O4	108 (2)	O2—C8—H8B	109.2
C2—C1—C6	120.9 (2)	C7—C8—H8B	109.2
C2—C1—H1A	119.5	H8A—C8—H8B	107.9
C6—C1—H1A	119.5	O3—C9—O2	121.8 (2)
C3—C2—C1	119.2 (2)	O3—C9—C10	126.4 (2)
C3—C2—H2A	120.4	O2—C9—C10	111.78 (19)
C1—C2—H2A	120.4	C15—C10—C11	118.4 (2)
C2—C3—C4	121.5 (2)	C15—C10—C9	122.44 (19)
C2—C3—Cl1	119.7 (2)	C11—C10—C9	119.07 (19)
C4—C3—Cl1	118.8 (2)	C12—C11—C10	120.6 (2)
C3—C4—C5	118.9 (2)	C12—C11—H11A	119.7
C3—C4—H4A	120.5	C10—C11—H11A	119.7
C5—C4—H4A	120.5	C11—C12—C13	120.35 (19)
C4—C5—C6	121.1 (2)	C11—C12—H12A	119.8
C4—C5—H5A	119.5	C13—C12—H12A	119.8
C6—C5—H5A	119.5	O4—C13—C12	118.0 (2)
C1—C6—C5	118.5 (2)	O4—C13—C14	122.5 (2)
C1—C6—C7	118.9 (2)	C12—C13—C14	119.5 (2)
C5—C6—C7	122.6 (2)	C15—C14—C13	120.2 (2)
O1—C7—C6	122.0 (2)	C15—C14—H14A	119.9
O1—C7—C8	120.9 (2)	C13—C14—H14A	119.9
C6—C7—C8	117.15 (19)	C14—C15—C10	120.92 (19)
O2—C8—C7	111.9 (2)	C14—C15—H15A	119.5
O2—C8—H8A	109.2	C10—C15—H15A	119.5
			
C6—C1—C2—C3	−0.3 (3)	C8—O2—C9—O3	−0.4 (3)
C1—C2—C3—C4	−0.1 (4)	C8—O2—C9—C10	−178.72 (19)
C1—C2—C3—Cl1	179.18 (18)	O3—C9—C10—C15	−174.2 (2)
C2—C3—C4—C5	0.5 (3)	O2—C9—C10—C15	4.1 (3)
Cl1—C3—C4—C5	−178.78 (17)	O3—C9—C10—C11	3.2 (3)
C3—C4—C5—C6	−0.5 (3)	O2—C9—C10—C11	−178.51 (18)
C2—C1—C6—C5	0.3 (3)	C15—C10—C11—C12	−1.1 (3)
C2—C1—C6—C7	178.9 (2)	C9—C10—C11—C12	−178.6 (2)
C4—C5—C6—C1	0.1 (3)	C10—C11—C12—C13	0.9 (3)
C4—C5—C6—C7	−178.4 (2)	C11—C12—C13—O4	−179.6 (2)
C1—C6—C7—O1	3.7 (3)	C11—C12—C13—C14	0.4 (3)
C5—C6—C7—O1	−177.8 (2)	O4—C13—C14—C15	178.4 (2)
C1—C6—C7—C8	−174.6 (2)	C12—C13—C14—C15	−1.6 (3)
C5—C6—C7—C8	4.0 (3)	C13—C14—C15—C10	1.4 (3)
C9—O2—C8—C7	−73.7 (3)	C11—C10—C15—C14	−0.1 (3)
O1—C7—C8—O2	0.8 (3)	C9—C10—C15—C14	177.3 (2)
C6—C7—C8—O2	179.02 (18)		

### Hydrogen-bond geometry (Å, °)

**Table:**

*D*—H···*A*	*D*—H	H···*A*	*D*···*A*	*D*—H···*A*
O4—H1O4···O3^i^	0.78 (4)	2.01 (4)	2.783 (3)	168 (3)

Symmetry codes: (i) *x*, *y*−1, *z*.
